# Charles E. Esterly, D.D.S.

**Published:** 1901-10-15

**Authors:** 


### OBITUARY.

### CHARLES E. ESTERLY, D.D.S.

The following is taken from the September number of the *Western Dental Journal*, and pays a merited tribute to one well known to many of our readers. Wherever Dr. Esterly was known he was respected and honored for his unselfish devotion to the higher interests of the profession. His death seems untimely, and his genial countenance will be missed at coming gatherings of professional men.

Dr. Esterly, of Lawrence, Kansas, died Tuesday morning, September 10th, at 11 o'clock A. M. His many friends were profoundly shocked upon receiving the sad news. The deplorable event came unheralded. Dr. Esterly had some slight symptoms of impeded circulation as early as Saturday preceding his death, but was about, and on Monday attended to his practice until 3 o'clock, when he and Mrs. Esterly took a drive and witnessed the football practice on McCook Field. Early in the evening the paralysis seemed to extend from the fingers to the arms, and for treatment massage and hot baths were given. No apprehension of grave results was felt, and he retired expecting to be about next day as usual. About 4 o'clock Monday morning more serious conditions presented, the paralysis extending over the body. Physicians were hastily summoned, but all treatment proved useless, and he gradually sank until the end came at 11 o'clock. He conversed with Mrs. Esterly and others until 9:30, was perfectly conscious and rational, declared he suffered absolutely no pain, knew the end was approaching, and so went peacefully to his long rest.

The cause of death is given by his physicians as blood-clot at base of brain.

Charles E. Esterly was born at Columbiana, Ohio, July 5, 1863; graduated in the public schools in May, 1881, and entered Ohio State University in September of the same year, where he remained one year. His appreciation of his opportunities at that institution was very keen and one of the pleasant experiences of his life, to which he often referred. At even that age he felt that the student received only so much as he gave to himself.

In the autumn of 1882 he entered the dental office of Dr. J. E. Whinnery, of Salem, Ohio, where he received the foundation of the ethical ideas that always characterized him in his profession. In October, 1883, he matriculated at the Ohio College of Dental Surgery.

In the spring of 1884, when Dr. Patterson was about to leave Lawrence, Kansas, for Kansas City, Mo., Dr. Esterly was his successor. On March 28, 1884, he arrived at Lawrence. In the fall of 1885 he returned to Cincinnati and completed his course, and graduated on March 4, 1886, returning to Lawrence shortly afterward. On October 25, 1887, he was married to Lena L. Hayden, at Columbiana, Ohio, who survives him.

Dr. Esterly was a prominent member of the Kansas State Dental Association, of which he was President in 1894; of the Missouri State Dental Association, and of the Delta Sigma Delta dental fraternity. At the time of his death he was deputy master of Nu Chapter of Delta Sigma Delta in the Kansas City Dental College, and was the organizer of the same. He has been a teacher in the Kansas City Dental College since the year 1892. Dr. Esterly was a member of many organizations other than dental. He was an exalted ruler of the Elks, a thirty-second-degree Mason, a Mystic Schriner, a Knight of Phythias, and a member of the college fraternity Phi Kappa Pi. He was thirty-eight years of age.

At the funeral the attendance was very large, and prominent dentists from Kansas and Missouri came in large numbers with their sympathy to Dr. Esterly's family, and to testify to their appreciation of the deceased. The services at the funeral were very simple, and were conducted by Dr. Cordley, of the Congregational Church. Dr. Hungerford, of Kansas City, also made a short address, and Mrs. Crane, of Kansas City, sang "One Sweetly Solemn Thought." The pall-bearers were Dr. C. B. Reed, of Topeka; Dr. J. P. Root, of Kansas City; Dr. C. L. Hungerford, of Kansas City; Dr. S. J. Rentz, of Leavenworth; Dr. R. A. Wasson, of Ottawa; Dr. Edward Bumgardner, of Lawrence; Dr. A. H. Thompson, of Topeka; Dr. J. D. Patterson, of Kansas City.

The writer knows of no one who in so short a time has risen to prominence in his profession, and who in that time has become more widely known and loved for his many personal qualities, Outside of his home, he was never so happy as when among kindred spirits of the dental profession discussing points in theory and practice. Especially can it be said of Dr. Esterly that he has been a power in elevating the ethical standards of his profession, and although his friends may not meet him about this earth, yet his memory and his work will have its good influence upon all who have met him and upon the profession generally. No more shall we meet this genial comrade, and our hearts rebel; the times seem out of joint. Oh, how we shall miss him! Death is so genuine a fact that it excludes falsehood. It is a touch-stone that proves the gold and disproves the baser metal. So we do not oversay when we laud our dead friend. Death gives a truer idea of his character than we possessed while he was living amongst us. Fare well, citizen, companion, friend. Good-night, "till in some brighter clime we say, Good-morning.—*J. D. P.*

